# Assessing Vulnerability to Surges in Suicide-Related Tweets Using Japan Census Data: Case-Only Study

**DOI:** 10.2196/47798

**Published:** 2023-08-10

**Authors:** Toshiharu Mitsuhashi

**Affiliations:** 1 Center for Innovative Clinical Medicine Okayama University Hospital Okayama Japan

**Keywords:** case-only approach, mass media, public health, social media, suicidal risk, suicide prevention, suicide, suicide-related tweets, Twitter

## Abstract

**Background:**

As the use of social media becomes more widespread, its impact on health cannot be ignored. However, limited research has been conducted on the relationship between social media and suicide. Little is known about individuals’ vulnerable to suicide, especially when social media suicide information is extremely prevalent.

**Objective:**

This study aims to identify the characteristics underlying individuals’ vulnerability to suicide brought about by an increase in suicide-related tweets, thereby contributing to public health.

**Methods:**

A case-only design was used to investigate vulnerability to suicide using individual data of people who died by suicide and tweet data from January 1, 2011, through December 31, 2014. Mortality data were obtained from Japanese government statistics, and tweet data were provided by a commercial service. Tweet data identified the days when suicide-related tweets surged, and the date-keyed merging was performed by considering 3 and 7 lag days. For the merged data set for analysis, the logistic regression model was fitted with one of the personal characteristics of interest as a dependent variable and the dichotomous exposure variable. This analysis was performed to estimate the interaction between the surges in suicide-related tweets and personal characteristics of the suicide victims as case-only odds ratios (ORs) with 95% CIs. For the sensitivity analysis, unexpected deaths other than suicide were considered.

**Results:**

During the study period, there were 159,490 suicides and 115,072 unexpected deaths, and the number of suicide-related tweets was 2,804,999. Following the 3-day lag of a highly tweeted day, there were significant interactions for those who were aged 40 years or younger (OR 1.09, 95% CI 1.03-1.15), male (OR 1.12, 95% CI 1.07-1.18), divorced (OR 1.11, 95% CI 1.03 1.19), unemployed (OR 1.12, 95% CI 1.02-1.22), and living in urban areas (OR 1.26, 95% CI 1.17 1.35). By contrast, widowed individuals had significantly lower interactions (OR 0.83, 95% CI 0.77-0.89). Except for unemployment, significant relationships were also observed for the 7-day lag. For the sensitivity analysis, no significant interactions were observed for other unexpected deaths in the 3-day lag, and only the widowed had a significantly larger interaction than those who were married (OR 1.08, 95% CI 1.02-1.15) in the 7-day lag.

**Conclusions:**

This study revealed the interactions of personal characteristics associated with susceptibility to suicide-related tweets. In addition, a few significant relationships were observed in the sensitivity analysis, suggesting that such an interaction is specific to suicide deaths. In other words, individuals with these characteristics, such as being young, male, unemployed, and divorced, may be vulnerable to surges in suicide-related tweets. Thus, minimizing public health strain by identifying people who are vulnerable and susceptible to a surge in suicide-related information on the internet is necessary.

## Introduction

The internet and social media have recently become important sources of health-related information [1,2], wherein the importance of suicide prevention has also been highlighted [2]. Suicide prevention among young people, who spend a significant amount of time on the internet and social media, is a related concern [3] leading to several recent studies on the subject [4,5].

Recent evidence suggests that reporting suicide through mass media has influenced trends in suicide deaths. Indeed, several researchers are concurrently studying the effect of conventional mass media on mental health [6-8] as well as the occurrence of copycat suicides resulting from mass media reporting [9-12]. To avoid the increased risk caused by these reports, the World Health Organization has issued recommendations for media coverage of suicides [13].

In this regard, social media could play an important role in disseminating appropriate information regarding suicide [14-17]. Consequently, examining the effects of social media on suicide risk has become a crucial public health concern. For instance, Fahey et al [18] demonstrated the role of emotional responses in social media messages concerning celebrity suicides and their correlation with subsequent increases in national suicide rates. Miyagi et al [19] highlighted the potential for social media–based prevention measures to be undermined by the proliferation of offensive opinions and nonpreventive advertisements following a high-profile suicide-related incident. Numerous other studies have corroborated the association between social media use and suicide risk, further emphasizing the need for a comprehensive understanding of this complex relationship [20-24]. Nonetheless, regulating suicide-related information on these platforms is challenging due to the vast number of users and limited oversight.

As with other causal relationships [25-27], heterogeneity may exist in social media’s causal effect on suicide risk. This indicates that there are subpopulations with increased risk of exposure (vulnerable populations) and those with unchanged or reduced risk. Thus, focusing preventive measures on vulnerable populations is advantageous. In this context, identifying individual attributes predisposing individuals to the influence of suicide-related content enables the implementation of preventive strategies against suicide and provides a better guide for resource allocation.

The mechanism by which individual attributes cause differences in vulnerability may have both biological and contextual effects (Textbox 1) [28]. Although it is difficult to estimate the contribution of each effect, it is useful to identify the vulnerability caused by the combination of these effects to consider public health measures.

Using appropriate data, such vulnerability can be estimated as a regression coefficient on the interaction term by inputting a statistical model with suicide as the response variable as well as social media and individual characteristics of interest and their interactions as explanatory variables [26]. However, because of the difficulty of preparing adequate data, at present, little is known about interactions between social media and personal characteristics on suicide risk.

This study aims to identify the factors underlying vulnerability to suicide resulting from sharp increases in suicide-related posts on Twitter. Twitter data are relatively easy to access, and tweets can be used as a surrogate marker for information diffusion within popular social media platforms. To accomplish this, a case-only analysis was conducted, as detailed below.

**Possible mechanisms for differences in vulnerability based on personal characteristics.**

Vulnerability is assumed to occur when these two effects differ by individual characteristics.

**Biological effect**

(1) Actual exposure.

In other words, viewing suicide-related tweet.

What are the characteristics of individuals who view suicide-related tweets?

(2) Risk is increased by direct exposure.

What individual attributes increase the risk of suicide by viewing suicide-related tweets?

**Contextual effect**

(3) Risk is increased by circumstances surrounding the person influenced by exposure.

What individual characteristics increase suicide risk owing to surrounding circumstances (eg, unmeasured self-harm in the neighborhood posted on social media), other than viewing suicide-related tweet viewing?

Biological effects cannot occur without actually viewing suicide-related tweets.

Contextual effects can occur without actually viewing suicide-related tweets.

## Methods

### Study Design

The case-only design, originally used to examine gene-environment interactions, was recently proposed by Armstrong [29] to identify interactions due to time-varying exposure. Schwartz [30] presented an applied study of a case-only approach, which is specifically referenced in this study. There is detailed information on these methods in the original works of literature [29,30].

This study used Japanese census data on suicides to calculate case-only odds ratios (ORs), enabling the identification of personal characteristics that make individuals more vulnerable to suicide due to a surge in suicide-related tweets.

The case-only approach in this study is outlined below. Here is an example of the interaction between suicide-related tweet surges (0=no surge day and 1= surge day) and sex (0=female and 1=male). First, daily instances of suicide (Yt) can be modeled as a Poisson regression (equation 1), where *Surge* represents an indicator variable for suicide-related tweet surge day. *Others* denote seasonal terms and time-varying risk factors, which have become increasingly complex.

**(1)**



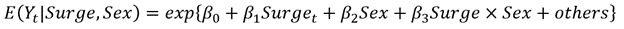



The expected number of people who die by suicide is calculated using equations 2 and 3 for females with Surge=0 and Surge=1, respectively:

**(2)**



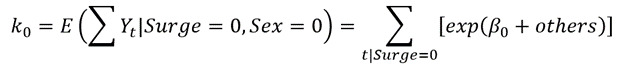



**(3)**



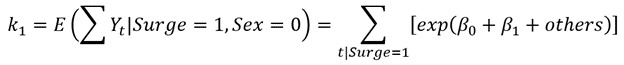



For males (sex=1), equations 4 and 5 are used for Surge=0 and Surge=1, respectively:

**(4)**



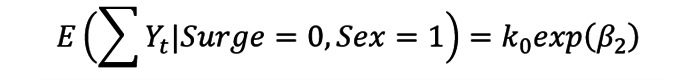



**(5)**



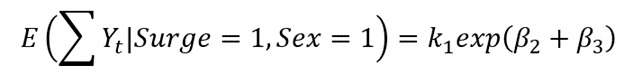



By using these equations, as in Table 1, a 2-by-2 table of suicide counts by tweet surge and sex can be constructed, yielding an OR for the interaction of exp(β3), as illustrated in equation 6.

**(6)**



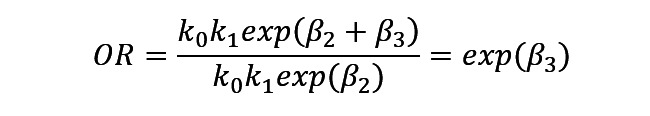



This analysis corresponds to a logistic regression among individuals who died by suicide (equation 7), predicting sex with *Surge* as the exposure.

**(7)**



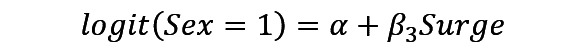



A logistic regression model predicting sex as a function of tweet surge yields similar results as the Poisson model without requiring complex time-varying covariates. If the analysis shows a significant interaction between sex and tweet surge, it indicates differing risks between male and female individuals. Although the case-only analysis uses individuals who died by suicide, the inference still applies to the entire population.

In summary, in this study, a 2 by N table, like Table 1, was created for environmental level exposure (tweet surge) and individual characteristics, and the ORs were calculated using a logistic regression model. As presented in equation 6 above, this allowed us to calculate the exponents of the coefficients of the interaction terms. This study design differs significantly from the general study design, which examines the causal effects of exposure on outcomes. More specific research methods are discussed in the subsections that follow.

**Table 1:** Expected distribution of total deaths due to suicide.

Tweet surge	Female	Male
Nonsurge day	*k*_0_	*k*_0_*exp*(β_2_)
Surge day	*k*_1_	*k*_1_*exp*(β_2_+β_3_)

### Data Collection

#### Mortality Data

Mortality data were obtained from Japanese demographic statistics released by the Japanese Ministry of Health, Labor, and Welfare from January 1, 2011, to December 31, 2014. The data included information on sex, date of death, address code, marital status, occupation, cause of death based on the International Classification of Diseases (ICD)–10 code, and age at death. The data obtained for the cause of death were within the ICD-10 code range of W00-Y59. Although X60-X84 corresponded to “suicide,” the primary outcome of this study, for sensitivity analysis, data on other external causes and unexpected deaths were also obtained (W00-X59, Y01-Y59). The following personal characteristics were used as potential interaction variables: age at death, sex, occupation, marital status, and area of residence (urban or rural).

#### Twitter Data

Tweets were extracted from the period between January 1, 2011, and December 31, 2014, using a commercial service provided by Hotto Link Inc [31]. Approximately 10% of all tweets were provided as a random sample. The search keywords were as follows: (1) tweets including either of the Japanese words for “suicide” or “self-death” and (2) tweets additionally including “prevention,” “avoidance,” “countermeasure,” “support,” “project,” “symposium,” “conference,” “panel discussion,” or “discussion.” The tweets found using (2) were subtracted from the number of tweets found using (1), and the results were termed as “suicide-related tweets,” with the number of tweets calculated daily. See Textbox 2 for details of the Japanese keywords used in the search.

In addition to regular tweets, Twitter has a “retweet” function used to share information, as well as “reply” and “mention” functions, all of which include the user’s name. All these tweets were also extracted. Tweet numbers are affected by external noise (changes in Twitter user numbers, problems with the Twitter system, etc), which was reduced using a method proposed by Sano and Takayasu [32]. The calculation to determine the increase ratio for the previous day was applied to the noise-eliminated tweet numbers. If the increase ratio of the previous day was at or above the 95th percentile, that day was categorized as an exposed day; when this ratio was less than the 95th percentile, it was termed an unexposed day.

**Summary of Japanese words used in the Twitter search.**

- Tweets containing words related to suicide: including “suicide (自殺)” or “self-death (自死)”
- Tweets containing words related to suicide prevention and conference: including “prevention (予防),” “avoidance (防止),” “countermeasure (対策),” “support (支援),” “project (プロジェクト),” “symposium (シンポジウム),” “conference (カンファレンス),” “panel discussion (パネルディスカッション),” or “discussion(議論)”
- The number of suicide-related tweets was defined as (1) the number of all tweets minus (2) the number of prevention tweets.

### Data Analysis

**Figure 1:**
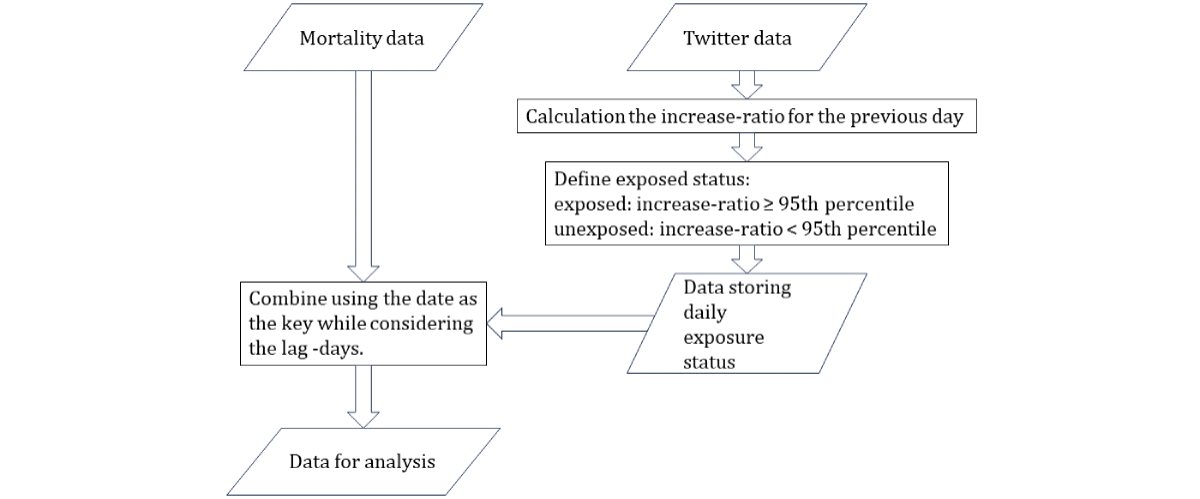
Manipulation flow of mortality data and Tweet data.

The data manipulation flow for the analysis is outlined in Figure 1. To create the data set for analysis, the mortality and tweet data sets (the exposure status was calculated) were combined using the date as a key. The date-keyed merging was performed by considering the lag days of 3 and 7 days. This procedure generated a data set that included each row’s candidate confounders and exposure status.

Descriptive statistics were used for the personal characteristics of the sample. The mean and SD of age at death were calculated.

For the number of suicide-related tweets, the distribution was calculated for the number of tweets, the difference from the previous day, and the ratio to the previous day. Furthermore, a time-series graph was created to visualize the variation in the number of tweets.

Age, sex, occupation, marital status, and area of residence at the time of death were selected as individual characteristics for analysis. Age was binarized into 40 or younger, as this was the age group assumed to be the most exposed to Twitter, and older than 40.

For personal characteristics of dichotomous variables (age, sex, and area of residence), using the data on time-independent information about the individual who died by suicide and time-dependent information about the number of suicide-related tweets, the logistic regression model was fitted with one of the personal characteristics of interest as a dependent variable and the dichotomous exposure variable defined in the Twitter data section above, that is, the indicator of the day when suicide-related tweets surged, as an independent variable. Case-only ORs and their 95% CIs were calculated as estimates of the interaction. This case-only OR can be interpreted as the magnitude of the interaction of personal characteristics on the effect of a tweet surge on suicide risk [29,30].

For personal characteristics of multinominal variables (job and marital status), to estimate the interaction between social media surge and the individual characteristics of interest, multinomial logistic regression analysis was performed to regress the multinominal personal characteristics on dichotomous social media surge, yielding relative risk ratios and their 95% CIs using the following as the base-level [33]; accordingly, “self-employed” for a job and “married” for marital status were used as the base-level. This model was selected since the dependent variable has more than 2 categories. The calculation of relative risk ratio is represented by equation 8, which is not exactly the same as OR but can be interpreted similarly to OR [29]. To circumvent notational complexity, both are represented as ORs hereafter.

**(8)**



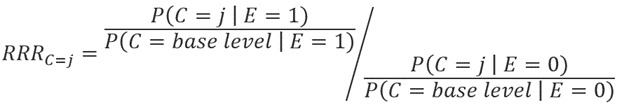



where *C* indicates the variable of a personal characteristic, *j* indicates one of the levels of the personal characteristic, and *E* indicates whether it is an exposure date, that is, *E=1* as exposed day and *E=0* as unexposed day.

Because analogous studies involving social media are limited, the study of meteorological conditions was used as a reference to determine the number of lag days. According to Schwartz [30], case-only ORs have been estimated with a lag of 0 day and 2 days. Since social media effects are not as biologically direct as meteorological conditions, with the assumption of a longer lag than meteorological events, this study used lags of 3 days and 7 days. In addition, because personal characteristics and suicide-related tweets were not associated with the base population, covariates were not used [34]. Any participants with missing values for age or occupation or a value of “other” in marital status were excluded from the analyses.

For the sensitivity analysis, data on unexpected deaths other than suicide were analyzed using the same method. All analyses were conducted using the statistical software Stata 14.2 (StataCorp).

### Ethical Considerations

The study protocol was reviewed and approved by the Ethics Committees of the Okayama University Graduate School of Medicine, Dentistry and Pharmaceutical Sciences and the Okayama University Hospital (approval numbers 914 and 1013). As the study was conducted using solely public information, obtaining consent from the study participants was deemed exempt in this context.

## Results

### Descriptive Statistics

During the study period, 159,490 suicides and 115,072 unexpected deaths occurred. Table 2 shows the deceased participants’ personal characteristics. Of the 159,490 people who died by suicide, 35,374 (22.2%) were aged 40 years or younger, and 103,893 (65.1%) were men. Of the 115,072 people who had unexpected deaths other than suicide, 6177 (5.4%) were aged 40 years or younger, and 63,594 (55.3%) were men.

**Figure 2:**
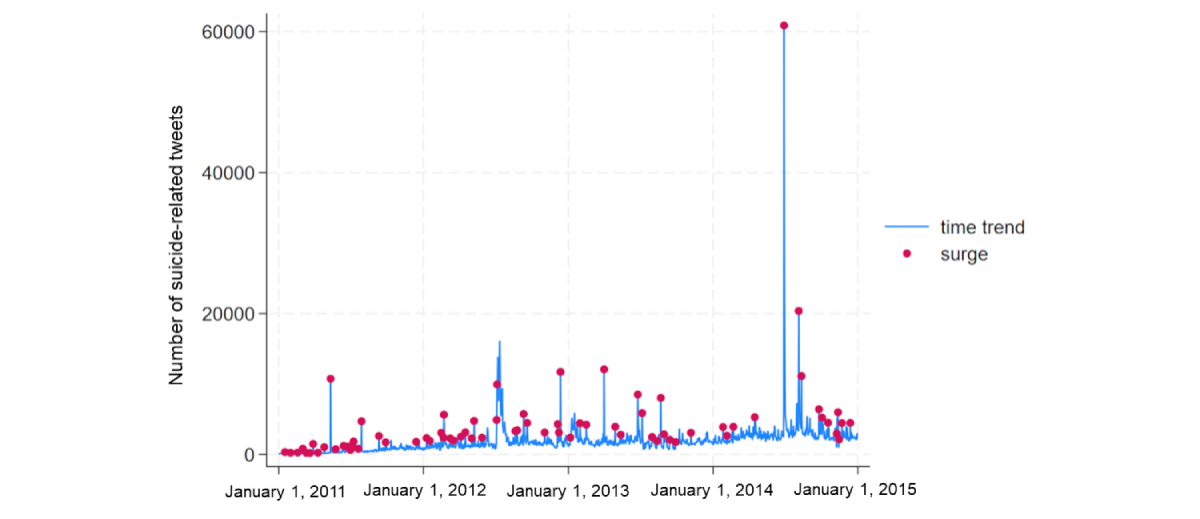
Time-series changes in the number of suicide-related Tweets.

The number of suicide-related tweets was 2,804,999. Table 3 shows the distribution of these tweets, previous day difference, and previous day ratio. The range was large, with a minimum of 57 and a maximum of 60,875 suicide-related tweets. The maximum ratio was 44 times that of the previous day, indicating a significant spike. A day with a previous-day ratio greater than 1.79, corresponding to the 95th percentile, was considered a surge day. The variation of the tweet is shown in Figure 2. The lines indicate time-series variation, and the dots denote exposure (a surge day). Days with a large day-to-day ratio are evenly distributed throughout the study period.

**Table 2:** Personal characteristics of people who died by suicide and had unexpected deaths in Japan, 2011-2014.

		Suicide (N=159,490)	Unexpected death (N=115,072)
**Age (years), mean (SD)**		58.5 (20.6)	75.5 (17.3)
**Age group (years), n (%)**			
	0-20	4607 (2.9)	1804 (1.6)
	21-40	30,767 (19.3)	4373 (3.8)
	41-60	43,983 (27.6)	10,526 (9.1)
	61-80	52,186 (32.7)	42,876 (37.3)
	≥81	26,583 (16.7)	55,169 (47.9)
	Missing	1364 (0.9)	324 (0.3)
**Sex, n (%)**			
	Male	103,893 (65.1)	63,594 (55.3)
	Female	55,597 (34.9)	51,478 (44.7)
**Occupation, n (%)**			
	Agriculture	9550 (6)	8701 (7.6)
	Self-employed	13,191 (8.3)	7040 (6.1)
	Corporate	34,472 (21.6)	14,987 (13)
	Other	17,312 (10.9)	8596 (7.5)
	Unemployed	66,639 (41.8)	64,682 (56.2)
	Missing	18,326 (11.5)	11,066 (9.6)
**Marital status, n (%)**			
	Married	65,500 (41.1)	50,548 (43.9)
	Unmarried	43,250 (27.1)	14,760 (12.8)
	Widowed	28,082 (17.6)	40,336 (35.1)
	Divorced	20,765 (13)	8823 (7.7)
	Other	1893 (1.2)	605 (0.5)
**Area, n (%)**			
	Urban	137,761 (86.4)	101,043 (87.8)
	Rural	21,729 (13.6)	14,029 (12.2)

**Table 3:** Distribution of the number of tweets, previous day difference, and ratio in Japan from 2011-2014.

	Minimum	5%	25%	50%	95%	Maximum
Suicide-related tweets	57	175	939	1637	4010	60,875
Previous day difference^a^	–34,714	–1127	–248.5	–16	1166	58,344
Previous day ratio^b^	0.14	0.59	0.83	0.98	1.79	44.3

^a^Previous day difference is defined as the number of tweets minus the number of tweets on the previous day.

^b^Previous day ratio is defined as the number of tweets divided by the number of tweets on the previous day.

### Estimates of Interaction for Personal Characteristics

Table 4 presents the case-only ORs calculated as interaction estimates. In the analysis of suicide, significant interactions were observed for all personal characteristics that were analyzed. In the analysis of the 3-day lag, individuals aged 40 years or younger had significantly higher interactions (OR 1.09, 95% CI 1.03-1.15) compared with older people. Additionally, participants who were male (OR 1.12, 95% CI 1.07-1.18), unemployed (OR 1.12, 95% CI 1.02-1.22), divorced (OR 1.11, 95% CI 1.03-1.19), and residents of urban areas (OR 1.26, 95% CI 1.17-1.35) had significantly higher interactions. Widowed individuals had a significantly smaller interaction when compared to those who were married (OR 0.83, 95% CI 0.77-0.89). Except for unemployment, significant relationships were also observed for the 7-day lag.

**Table 4:** Estimated interaction (case-only odds ratio [OR]) by personal characteristics of the individuals who died by suicide.

		3-day lag, case-only OR (95% CI)^a^	7-day lag, case-only OR (95% CI)^a^
**Age**			
	40 years or younger	1.09 (1.03-1.15)^b^	1.06 (1.01-1.12)^b^
**Sex**			
	Male	1.12 (1.07-1.18)^b^	1.12 (1.07-1.18)^b^
**Occupation**			
	Agriculture	0.99 (0.87-1.13)	1.09 (0.96-1.24)
	Corporate	1.02 (0.92-1.12)	1.01 (0.92-1.11)
	Other	1.07 (0.95-1.19)	1.05 (0.94-1.17)
	Unemployed	1.12 (1.02-1.22)^b^	1.08 (0.98-1.18)
**Marital status**			
	Unmarried	1.04 (0.98-1.10)	1.06 (1.00-1.12)
	Widowed	0.83 (0.77-0.89)^b^	0.90 (0.84-0.97)^b^
	Divorced	1.11 (1.03-1.19)^b^	1.16 (1.08-1.25)^b^
**Area**			
	Urban	1.26 (1.17-1.35)^b^	1.27 (1.18-1.37)^b^

^a^The reference categories are as follows: age: >40 years; sex: female; occupation: self-employed; marital status: married; area: rural.

^b^*P*<.05.

### Sensitivity Analysis

Table 5 presents the results of the sensitivity analysis. Apart from marital status, there were no significant interactions for the sensitivity analyses of unexpected deaths other than suicide. With regard to marital status, the widowed had a significantly larger interaction than those who were married (7-day lag: OR 1.08, 95% CI 1.02-1.15). However, for the 3-day lag, there were no significant interactions.

**Table 5:** Estimated interaction (case-only odds ratio [OR]) by personal characteristics of the individuals having unexpected deaths.

		3-day lag, case-only OR (95% CI)^a^	7-day lag, case-only OR (95% CI)^a^
**Age**			
	40 years or younger	1.05 (0.93-1.18)	1.04 (0.93-1.17)
**Sex**			
	Male	1.02 (0.96-1.07)	0.97 (0.92-1.02)
**Occupation**			
	Agriculture	0.89 (0.78-1.03)	0.98 (0.85-1.13)
	Corporate	0.94 (0.83-1.07)	0.99 (0.87-1.13)
	Other	0.87 (0.75-1.00)	0.91 (0.79-1.06)
	Unemployed	0.92 (0.82-1.02)	0.97 (0.87-1.09)
**Marital status**			
	Unmarried	1.04 (0.95-1.13)	1.04 (0.95-1.13)
	Widowed	1.00 (0.94-1.06)	1.08 (1.02-1.15)^b^
	Divorced	1.08 (0.98-1.20)	0.96 (0.86-1.07)
**Area**			
	Urban	1.00 (0.92-1.09)	1.05 (0.97-1.14)

^a^The reference categories are as follows: age: >40 years; sex: female; occupation: self-employed; marital status: married; area: rural.

^b^*P*<.05.

## Discussion

### Principal Results

This study reveals interactions between the surge in suicide-related tweets and individual characteristics such as sex, job, marital status, and area. It also found nearly no interactions for unexpected deaths other than suicide. Thus, these interactions appear to be specific to suicide. This study used tweets as a surrogate marker for information diffusion across social media platforms. If there were similar surges in suicide-related information on other social media platforms, the findings could be generalized to platforms other than Twitter.

Despite the small size of the interaction’s point estimate, the public health implications of this interaction can fluctuate depending on the circumstances. To elaborate, the greater the impact of exposure to suicide-related tweets, the baseline risk among those not exposed, and the larger the population affected, the more substantial the public health impact of the interaction becomes, even if the size of the interaction’s point estimate is small. Regrettably, this study could not conclusively examine these factors. However, in light of the recent surge in social networking service users, it is posited that this issue will garner increasing significance in the future.

### Interpretations of Results

The reason for this vulnerability may be due to 2 effects, as shown in Textbox 1. First, there may be a biological effect. Individuals with some characteristics are more likely to view suicide-related tweets, and when they do, they feel strong psychological stress, which increases their suicide risk. Second, a contextual effect is also possible. This is an effect in which psychological stress is increased by the surrounding situation (eg, neighborhood self-harm), not through direct viewing of the tweet, and suicide risk is increased. The characteristics that had a high vulnerability in this study are likely to be susceptible to these effects.

Although it was not possible to determine the extent to which each of these factors contributed to vulnerability in this study, external information implies that the frequency of social media visits may contribute to vulnerability, as discussed in the following paragraphs.

The results showed an association between increased suicide-related tweets and a higher susceptibility to suicide in people aged 40 years or younger. This age group accounts for the largest proportion of daily Twitter users [35]. Thus, these individuals are more likely to be exposed to suicide-related information through social media platforms. Furthermore, during surges in suicide-related tweets, men had a higher vulnerability to suicide than women did. It has been reported that in Japan, compared to male suicides, women engage in more self-injurious behaviors before a nonfatal or fatal suicide attempt [36]. Women may be at increased risk for suicide due to the escalation of these self-harming behaviors. If so, they may be more strongly influenced not by suicide-related tweets but by other factors that encourage self-harm (eg, social media information that leads to negative self-evaluations regarding beauty, body shape, etc).

Unemployment indicated a higher interaction than self-employment. A white paper on suicide prevention in Japan [35] reported that 55.6% of those who died by suicide were unemployed. Unemployed people are more likely to die by suicide because of economic problems. Furthermore, they may have more opportunities to browse social media than working people with workplace-related restrictions. Thus, unemployed individuals may be more susceptible to surges in suicide-related information.

The widowed had a lower interaction than married people, whereas divorcees had a higher effect. Previous studies on marital status and suicide deaths have reported a greater suicide risk among divorcees and the widowed compared with married individuals [37-39]. These findings may explain why suicide risk is more likely to increase among divorced than married individuals during a surge in suicide-related tweets. In addition, this study’s finding of lower vulnerability among the widowed is inconsistent with previous results. This may indicate that the widowed are generally older and less likely to be exposed to social media information.

Urban residents had a higher interaction when compared to rural residents. Although internet usage in Japan is increasing, users are still highly concentrated in urban areas [40]. Thus, more opportunities to use social media likely lead to higher interactions for urban residents.

Based on the results of this study, individuals who frequently use social media seem to have a positive interaction on suicide. Recent studies have also reported that increased social media exposure leads to mental health problems [41]. Therefore, reducing social media exposure may be a necessary suicide prevention measure. In particular, when suicide-related information is expected to increase rapidly owing to sensational news, attention should be paid to reducing the amount of exposure.

By contrast, the current results might simply reflect the fact that suicidality-prone individuals tend to cluster together before a negative event. In other words, individuals who are already vulnerable to suicide tend to cluster together owing to their vulnerability, and the suicide risk may be increased in this cluster when negative events occur [42]. In this study, suicide-vulnerable individuals may have become clustered by following and retweeting similar accounts even before the surge in suicide-related tweets. If individuals with the characteristics that have positive interactions shown in this study were clustered even before the surge in suicide-related tweets, identifying these clusters and providing suicide-preventive interventions would be useful. In recent years, machine learning has been used to predict suicide risk [43-45], and further research has identified clusters with high suicide risk.

Additionally, Twitter and other social media sites sometimes serve as platforms for spreading false information [46]. It has been reported that fake news could also potentially cause mental health problems [47]. It is necessary to improve health literacy in order to identify and separate false information from correct health information. To improve health literacy, training [48] and accurate information must be made readily available to users [1,49-52]

### Strengths and Limitations

This is the first study to clarify the relationship between social media and suicide risk. These findings could become a substantial knowledge base when considering future suicide prevention measures. As official Japanese demographic statistics were used, the data are highly valid. In addition, because a 10% random sample of tweets was used, the data collected are relatively unbiased. Thus, the data have high reliability, and a large sample size was considered. At 35 million people, Japan has a high number of active monthly Twitter users [53], with a high user percentage of 31% [54]. Thus, Twitter is a valid surrogate marker, representative of social media as a whole.

This study had some limitations. First, personal characteristics that were unavailable in the Japanese demographic census could not be investigated. Second, interaction was evaluated using multiplicative measures. An additive measure would have been preferable for public health purposes but was not computable because of the study design. Third, it is possible that the tweets were not collected in their entirety. In other words, some tweets related to suicide might have been missing. Additionally, while it is thought that specific usage times could impact interactions, no data exist regarding internet or social media usage times. Finally, the correlations between mass media reports and tweet numbers were not investigated.

### Conclusions

Using a case-only analysis, this study identified the personal characteristics that have a modifying effect on suicide based on surges in suicide-related tweets. The following attributes were positive interactions: being male, divorced, unemployed, an urban resident, and 40 years of age or younger. It is necessary to minimize public health strain by identifying people who are vulnerable and susceptible to a surge in suicide-related information on the internet.

## Abbreviations

ICD: International Classification of Diseases
OR: odds ratio

## Data Availability

This study uses Japanese mortality data and the daily suicide-related tweet count. Based on these data, the data set containing mortality cannot be published. These mortality data were acquired in accordance with Article 33 of the statistical law of Japan. These data are forbidden from being disclosed. The Ministry of Internal Affairs of Japan has released “Guidelines on operation of Article 33 of the statistical law” [55]. This guideline states that “publication of acquired data is not allowed” (page 17, line 4). Meanwhile, daily suicide-related tweet count data are available on Zenodo [56].
